# Traffic offending and deterrence: An examination of recidivism amongst drivers in Victoria, Australia born prior to 1975

**DOI:** 10.1371/journal.pone.0239942

**Published:** 2020-10-01

**Authors:** Hayley McDonald, Janneke Berecki-Gisolf, Karen Stephan, Stuart Newstead

**Affiliations:** Monash University Accident Research Centre, Monash University, Clayton, Victoria, Australia; Tsinghua University, CHINA

## Abstract

To deter the performance of illegal driving behaviours, traffic infringement notices may be issued. Whilst there is a substantial body of research that has examined rates of reoffending following a traffic infringement, there have been few studies examining the length of time to next traffic offence. Where this research has been conducted, the findings do not provide current understandings, given the substantial changes in traffic sanctioning over time. The aim of this study was to address this gap, by examining risk factors for recidivism following a driver receiving a traffic infringement notice, as well as the time to next traffic offence. Licensing and infringements data held in the Driver Licensing System (DLS), maintained by the road authority in Victoria, Australia were used. All drivers included in the study were born prior to 1975, and received their first Victorian drivers licence between 1994 and 2016. Data from 203,620 drivers were used. Cox proportional hazards modelling was undertaken to examine factors associated with recidivism within 12 months of receiving a traffic infringement. 131,691 (64.7%) drivers had received at least one traffic infringement in Victoria, Australia since receiving their Victorian driver’s licence. Factors found to be associated with longer time to further traffic offending in the year that followed the first infringement included being female; receiving a first Victorian driver’s licence when aged 45+ years; and being licenced 10+ years. Traffic infringements deter some groups of Victorian drivers, but not others. If drivers are to be deterred from further illegal driving behaviour, it is important other countermeasures are developed and trialled.

## Introduction

Road trauma is a significant global public health issue. In 2016, 1.35 million people died globally as a result of road trauma [1]. Factors associated with the incidence of trauma on the roads include road and roadside design and condition [2–5], vehicle design and condition [3, 5–8] and the environment [3, 5]. Human behaviour and error [3, 5, 9–12], which includes the performance of illegal driving behaviours, has also been found to be associated with increased risk of being involved in a road crash [13]. Speeding [14, 15], drink driving [16], drug driving [17] and mobile phone use whilst driving [18–20] are all associated with an increased occurrence of road crashes.

In many jurisdictions globally, drivers who are found to have performed an illegal driving behaviour receive a sanction that seeks to deter them from performing similar driving behaviours again [21]. Thus, punishments issued to drivers who perform illegal behaviours on the roads are consistent with the theory of specific deterrence. This theoretical perspective is based on the assumption that, after receiving a sanction for an illegal behaviour, a person will avoid the same pattern of behaviour in the future, to avoid further sanctions [22–27]. In the Australian state of Victoria, people who are detected by police or a road safety camera driving in an illegal manner will receive a traffic infringement notice. In most cases, this requires the driver to pay a fine, and demerit points are placed on their licence (which can ultimately result in licence suspension if a threshold is exceeded). The value of the fine and the number of demerit points issued is dependent upon the severity of the offence [28–30].

The significant body of research exploring specific deterrence, recidivism and traffic offending has primarily focused on drink driving [31–37] and speeding [38]. Much less is known about recidivism and traffic offending more broadly, as well as the many other types of offences that drivers may perform on the roads. Furthermore, the majority of studies have only quantified rates of recidivism, without considering the length of time following a traffic infringement that drivers are deterred from further offending. One exception is a study by Watson, Siskind, Fleiter, Watson and Soole, who, amongst other areas of analysis, examined time to reoffence following changes in speeding sanctions in the Australian state of Queensland [39]. They found that rather than time to reoffence increasing in length following the introduction of harsher sanctions, the time to a subsequent speeding offence actually decreased [39]. A second exception is a study by Haque, who applied a statistical model to examine the effectiveness of the demerit points system in Victoria, Australia [40]. Haque evaluated the time between drivers’ first and second driving offences and second and third driving offences, to examine differences that could be attributed to the demerit point system [40]. It was found that the length of time between the second and third driving offences was longer than the length of time between the first and second, adjusting for the additional driving experience drivers had accumulated [40]. It was thus concluded that the demerit point system could be credited with achieving deterrence, by increasing the period of time between drivers offending behaviour [40].

Much has changed in the approximately 30 years since the Haque study was published (notably the data used by Haque was for driving offences between 1982 and 1985) [40], and these changes may have had an influence on deterrence, recidivism and the time between infringements. Melbourne, which is Victoria’s capital city, has undergone a substantial rise in population numbers, and this rise continues to take place [41]. In addition to growth in the inner city and suburbs, growth has taken place in outer urban areas [41]. As would be expected in times of substantial population growth, traffic volumes also inevitably increase, meaning the number of people using the roads today is greater than the numbers seen 30 years ago. Automated enforcement, including the use of fixed red light and speed cameras, as well as mobile speed cameras is also widely used in Victoria today [42]. Indeed, in recent years, as technology has continued to be upgraded and improved, there has been a substantial increase in the number of infringements being issued as a result of automated cameras capturing dangerous driving behaviour [42]. The number of behaviours that drivers can receive an infringement for has also increased. For example, 30 years ago, mobile phones were not widely used, and therefore, the laws for using phones whilst driving that are in place today were not in operation at the time of the Haque study, with laws coming into effect in 1999 [43]. The severity of sanctions has also increased over time. Drivers today can expect to receive fines that are of a greater monetary value and with a greater number of demerit points than would have been issued at the time of the Haque study.

The aim of the current study was therefore to examine factors associated with time until re-offending amongst drivers licenced to drive a car, in the current Victorian system of enforcement. This research identified whether infringements for traffic offences are an effective means of deterring illegal driving behaviours, through examining driver-related factors that may be associated with recidivism, as well as the effect of demerit points on subsequent traffic offending.

## Materials and methods

### Data source

Licensing and infringements data held by VicRoads (the Driver Licensing System (DLS)) were used. The Monash University Human Research Ethics Committee approved the research (approval number 2017-9868-13714). The data were analysed anonymously, so consent was not obtained from individuals. VicRoads is the government authority in Victoria, Australia, with responsibility for administering the licensing system [44]. The DLS contains information on all drivers who have ever held a Victorian drivers’ licence. Variables in the data set include demographics, licence history and infringement history. The variables contained in the DLS used in this study were:

- sex;
- date of birth;
- date of death (where applicable);
- date Victorian driver’s licence was first obtained;
- dates where a licence was cancelled, disqualified, expired, suspended, surrendered or void;
- licence type;
- dates of any traffic infringements received;
- type of offences for which traffic infringements were received;
- number of demerit points issued for each traffic offence;
- number of accumulated demerit points.

Due to changes in the way in which information was held in the Victorian DLS, complete records are only available for drivers first licensed since 8 July 1994. In the data extract available for this study, complete and accurate licensing records were available through to 21 May 2016; this was therefore the end date of the study.

The data extract used for this study only included records of drivers born on or prior to 31 December 1974. This meant the data available only included information on drivers who were at least 19.5 years old when they obtained their drivers licence. In Victoria, a driver’s licence can be obtained at the age of 18. Thus, the drivers in this data extract for whom complete licensing information was available obtained their licence at a slightly older age than the minimum age drivers are able to obtain a driver’s licence.

### Study sample selection

The following inclusion criteria were applied:

1. Never held an interstate licence (a licence from an Australian state other than Victoria) or overseas licence: the study was focused on the deterring influence of infringements in Victoria, and required drivers’ full licence history. Given full licence history was not accessible for drivers who had previously been licenced interstate and overseas, these drivers were not included.
2. Never received an infringement for a driving offence interstate: drivers who had ever received an interstate infringement were excluded from the study given the possible influence infringements received outside of Victoria could have upon driving within Victoria. It was not possible to determine if drivers had received any sanctions for driving offences committed overseas.

The first licence received after 8 July 1994 had to be a car licence. Drivers could still be included if they subsequently received another licence type (heavy vehicle or motorcycle) following their car licence. Drivers were not included if they had already held another licence type prior to obtaining their car licence, even if this licence was obtained after 8 July 1994.

### Statistical analyses

Drivers were stratified into two groups: 1) never received a traffic infringement; and 2) ever received a traffic infringement. Descriptive statistics (frequency and %) were used to compare these groups, in terms of sex, age at first licence and licence type.

The analyses then focused only on drivers who had been identified as having ever received a Victorian traffic infringement. Drivers’ infringements were numbered in order of occurrence, up to a maximum of six. In cases where drivers received more than one infringement on the same day (for example a speeding infringement and a red light infringement), the more serious offence was used as the main offence type and another variable was developed to indicate where multiple infringements were received on a single day. Offence severity was based upon number of demerit points issued.

The outcome of interest was receiving a subsequent traffic infringement within 12 months of the index offence. The timeframe of 12 months was selected based on previous research that suggested the deterrent effect of a traffic infringement would likely not continue to influence driver behaviour for greater than one year [45]. The index offence was defined as the type of offence (or most serious offence) on a single day. Drivers could have up to six index offences. Five separate analyses were undertaken to examine time to reoffending following the first, second, third, fourth and fifth offences.

Descriptive statistics were used to examine the median time between infringements for drivers who reoffended within one year. Cox proportional hazards models [46, 47] were developed to estimate the association between driver and infringement characteristics and reoffending in the 12 months that followed. Kaplan-Meier survival curves were used to visually assess proportionality of hazards [48].

Due to multicollinearity, it was not possible to simultaneously include offence type and demerit points issued to a driver in the same Cox proportional hazards model. Two separate series of Cox proportional hazards modelling were therefore developed, one for each measure.

As per the time to event approach, censoring was used in specified circumstances [49, 50]. First, drivers who did not reoffend within 12 months of an index infringement date were censored. The date of censoring was the final date of follow up (12 months post-infringement), or in cases where this date exceeded the final date of the study, the censoring date was 21 May 2016. Drivers were censored at their date of death (if applicable), where this death occurred within 12 months of them receiving an infringement for a driving offence.

Drivers who experienced licence loss as a result of their driving behaviour were excluded from the study at the point this licence loss occurred, even if they were later reissued with a drivers’ licence and received further infringements. Including drivers who had experienced licence loss would make it difficult to separate the effect of this sanction from the effects of receiving an infringement. Furthermore, keeping unlicensed drivers may result in an underestimation of the likelihood of reoffending as these drivers would most likely have a lower exposure to receiving a further infringement, as they would not have been driving if they were obeying the conditions of their licence loss.

Due to the offences of speeding at or more than 25km/h above the speed limit, drink driving and drug driving commonly leading to licence loss, drivers charged with these offences were excluded from the Cox proportional hazards modelling following this infringement. This decision was made due to concerns that the behaviour of the small number of drivers who did keep their licence following these offence types would not be representative of the subsequent driving behaviour generally of drivers following these offences. Drivers were included in all models prior to receiving an infringement for these offence types. For example, a driver whose first infringement was for mobile phone use whilst driving, their second infringement was for not stopping at a red light, their third infringement was for speeding at or more than 25km/h and their fourth infringement was for speeding at less than 10km/h above the speed limit, was still included in the modelling of their first and second infringements and second and third infringements. They were, however, excluded following their third infringement.

Drivers who experienced licence loss for reasons other than infringements were excluded from the study, but were introduced back into the study if they re-obtained their drivers licence and received further infringements. These drivers had been unlicensed for failing to renew their drivers licence on time or by reason of surrendering their licence, which can occur due to some health conditions. Unlike losing one’s licence by reason of driving behaviour, there was not any identifiable risk in reintroducing these drivers back into the study once they reobtained their driver’s licence.

### Variables included in the Cox proportional hazards models to examine their association with recidivism

The first series of Cox proportional hazards models included the following variables: sex, age at first licence, years licenced at index offence, licence type, demerit points on day of index offence, accumulated demerit points, and total offences on day of index offence. There were two separate demerit point variables. The first was demerit points on the day of the index offence, which was used for the first model only, as drivers did not have previous infringements. The second was accumulated demerit points, which was used in the second, third, fourth and fifth models: for these models, all drivers had previous offences. In Victoria, demerit points accumulate, and remain on a driver’s licence generally for three years following a driver receiving an infringement (there are some additional conditions for probationary drivers) [28]. If a driver exceeds 12 points in this three-year time period, they may lose their licence [28]. Thus, for these models, the accumulated demerit point variable was the sum of their index offence demerit points and any existing demerit points they had received in the three years prior.

In the second series of Cox proportional hazards models, sex, age at first licence, years licenced at index offence, licence type and total offences on day of index offence were again included. The second series differed from the first however, as the demerit point variables were excluded and the models were stratified by offence type. The decision to stratify by offence type was made given the offence type variable was found to be non-proportional: the hazard ratio (the relative hazard of reoffending for those who had committed different offences) was not constant over time, which violates the assumption of proportional hazards. Thus, it was necessary to take a different approach, with stratification by offence type identified as being most appropriate. Models were developed for six offence types to examine time until reoffending following the first, second and third index offences (speeding below 25km/h; failure to obey a traffic signal; failure to stop or give way; seat, seatbelt and helmet offences; overtaking, lane use and U-turn offences; and mobile phone offences). Due to low numbers of drivers, models were only developed for a subset of offence types following the fourth (speeding below 25km/h; failure to obey a traffic signal; seat, seatbelt and helmet offences and mobile phone offences) and fifth (speeding below 25km/h and failure to obey a traffic signal) index offences.

## Results

### Characteristics of the study population

In total, 203,620 drivers met the inclusion criteria. Table 1 presents the descriptive characteristics of these drivers, stratified by whether or not they had ever received a traffic infringement. Chi Square tests were used to examine the relationships between history of receiving a traffic infringement since obtaining a Victorian drivers licence and sex, age at first licence and licence type. A higher proportion of males than females had received at least one Victorian traffic infringement since obtaining their licence (difference of 4.8%, 95% CI 4.4–5.2%). Among drivers who obtained their drivers licence at an older age (45+ years), the proportion that had ever received an infringement for a traffic offence was relatively low. For example, 20.6 percent more drivers who received their first drivers licence before age 25 years had received at least one traffic infringement since obtaining their licence, compared to drivers who had received their licence age 45+ (95% CI 19.8–21.3%). Drivers who held a car licence only were the least likely to have ever received a traffic infringement, compared to drivers who held a combined car and heavy vehicle licence, who were the most likely to have ever received an infringement for a traffic offence, with a difference of 10.4 percent (95% CI 9.4–11.4%).

**Table 1:** Descriptive characteristics of study cohort based on traffic offending status.

Variable	Never received a traffic infringement	Ever received a traffic infringement	Total	*X*^2^ (df)	*p*
	**n (%)**	**n (%)**	**n (%)**		
Overall	71,929 (35.3)	131,691 (64.7)	203,620 (100.0)		
Sex				500.3 (1)	< .0001
Female	43,435 (37.4)	72,763 (62.6)	116,198 (57.1)		
Male	28,494 (32.6)	58,928 (67.4)	87,422 (42.9)		
Age at first licence				4560 (3)	< .0001
19.5–24	7,842 (28.2)	19,935 (71.8)	27,777 (13.6)		
25–34	22,283 (29.9)	52,264 (70.1)	74,547 36.6)		
35–44	23,998 (37.0)	40,817 (63.0)	64,815 (31.8)		
45+	17,806 (48.8)	18,675 (51.2)	36,481 (17.9)		
Licence type				572.0 (3)	< .0001
Car only	66,460 (36.1)	117,452 (63.9)	183,912 (90.3)		
Car and heavy vehicle	1,904 (25.7)	5,494 (74.3)	7,398 (3.6)		
Car and motorbike	2,636 (28.5)	6,602 (71.5)	9,238 (4.5)		
Car, heavy vehicle and motorbike	929 (30.2)	2,143 (69.8)	3,072 (1.5)		

### Median time between infringements: Driver and offence characteristics

Subsequent analyses only included drivers who had received at least one traffic infringement since obtaining their Victorian drivers’ licence. Table 2 provides the number of drivers who received subsequent infringements within 12 months of a previous infringement, and the median time to this next infringement. Following the first offence, 34,850 drivers (27.4%) received a second infringement within 12 months; following the second offence, 31,665 drivers (33.2%) received a further infringement within 12 months; following the third offence, 27,468 drivers (37.1%) received a further infringement within 12 months; following the fourth offence, 23,517 (40.3%) drivers received a further infringement within 12 months; following the fifth infringement, 19,939 (43.2%) drivers received a further infringement within 12 months. The total number of drivers reoffending was actually higher than the totals in Table 2, given only reoffending in the 12 months that followed each index offence was examined. Where a driver reoffended within 12 months, the median time to next infringement was consistently around four months. The exception was drivers who had held their licence for less than one year and in that time had received multiple infringements. The median time between the fifth and sixth infringements for this group was less than 1 month. This is to be expected however, as to receive this number of infringements within just one year of obtaining a driver’s licence would mean the offences would have been in close succession of one another. For those drivers with 7 or more accumulated demerit points, the time between their infringements in the following 12 months was less (between 3 and 4 months) when compared to drivers with a lower number of accumulated demerit points (over 4 months). Finally, where a driver received an infringement for more than one offence type on the day of the index offence, the median time to next infringement in the following 12 months was less than that observed for drivers who received an infringement for only a single offence on the day of the index offence (Table 2).

**Table 2:** Median time between traffic infringements received within 12 months of a previous infringement, by driver and offence characteristics.

Variable	One to two			Two to three			Three to four			Four to five			Five to six		
	Event (%)	Total	Median time (months)	Event (%)	Total	Median time (months)	Event (%)	Total	Median time (months)	Event (%)	Total	Median time (months)	Event (%)	Total	Median time (months)
Overall	34,850 (27.4)	127,246	4.56	31,665 (33.2)	95,377	4.44	27,468 (37.1)	74,064	4.20	23,517 (40.3)	58,327	4.11	19,939 (43.2)	46,199	4.08
Sex															
Male	17,252 (30.6)	56,322	4.44	15,300 (35.4)	43,178	4.32	13,141 (38.3)	34,273	4.20	11,348 (41.3)	27,473	4.18	9,779 (44.0)	22,242	4.11
Female	17,598 (24.8)	70,924	4.68	16,365 (31.4)	52,199	4.44	14,327 (36.0)	39,791	4.32	12,169 (39.4)	30,854	4.04	10,160 (42.4)	23,957	4.04
Age at first licence (years)															
18–24	5,248 (27.5)	19,116	4.68	5,228 (33.6)	15,565	4.32	4,849 (37.5)	12,930	4.32	4,380 (40.9)	10,721	4.27	3,879 (43.1)	8,990	4.08
25–34	13,761 (27.3)	50,389	4.68	13,193 (33.4)	39,517	4.56	11,880 (37.5)	31,668	4.32	10,358 (40.6)	25,487	4.08	8,973 (43.6)	20,601	4.11
35–44	11,278 (28.5)	39,568	4.44	9,513 (33.5)	28,422	4.44	7,843 (37.0)	21,186	4.20	6,488 (40.2)	16,131	4.04	5,305 (43.3)	12,264	4.08
45+	4,563 (25.1)	18,173	4.32	3,731 (31.4)	11,873	4.20	2,896 (35.0)	8,280	4.20	2,291 (38.3)	5,988	4.27	1,782 (41.0)	4,344	3.91
Years licenced at index offence															
Less than 1 year	8,351 (33.0)	25,320	4.32	2,288 (45.6)	5,022	3.72	673 (56.5)	1,192	2.76	224 (65.5)	342	1.89	87 (72.5)	120	0.85
1–4 years	14,955 (27.4)	54,577	4.68	12,483 (36.8)	33,893	4.32	8,150 (43.6)	18,702	4.08	4,938 (48.8)	10,122	3.75	2,847 (53.4)	5,334	3.42
5–9 years	7,675 (25.8)	29,757	4.56	10,289 (32.5)	31,683	4.56	10,144 (37.6)	26,997	4.34	8,950 (42.7)	20,983	4.24	7,258 (46.4)	15,647	4.08
10+ years	3,869 (22.0)	17,592	4.56	6,605 (26.7)	24,779	4.56	8,501 (31.3)	27,173	4.47	9,405 (35.0)	26,880	4.27	9,747 (38.8)	25,098	4.34
Licence type															
Car only	30,694 (27.0)	113,647	4.56	27,714 (32.8)	84,438	4.44	23,859 (36.7)	65,006	4.21	20,429 (40.2)	50,824	4.11	17,188 (43.0)	39,976	4.08
Car and heavy vehicle	1,646 (31.3)	5,263	4.32	1,625 (37.4)	4,342	4.32	1,440 (39.8)	3,617	4.54	1,254 (41.3)	3,038	4.22	1,129 (44.8)	2,519	4.04
Car and motorbike	1,921 (30.5)	6,292	4.32	1,774 (36.7)	4,973	4.20	1,646 (40.2)	4,092	4.34	1,405 (41.9)	3,353	4.08	1,221 (43.8)	2,788	3.91
Car, heavy vehicle and motorbike	589 (28.8)	2,044	5.04	552 (34.0)	1,624	4.54	523 (38.8)	1,349	4.73	429 (38.6)	1,112	4.11	401 (43.8)	916	4.31
Demerit points on day of index offence^a^															
One	20,950 (29.3)	71,479	4.44												
Two	527 (22.6)	2,327	4.68												
Three	13,045 (25.0)	52,119	4.80												
Four or more	328 (24.9)	1,315	4.44												
Accumulated demerit points^b^															
1–2				14,412 (34.1)	42,239	4.44	7,262 (35.4)	20,540	4.31	5,142 (38.3)	13,443	4.27	3,629 (39.8)	9,120	4.14
3–4				12,652 (32.3)	39,129	4.44	9,567 (36.1)	26,512	4.44	7,521 (38.7)	19,426	4.31	5,553 (41.1)	13,515	4.31
5–6				3,828 (31.9)	11,997	4.56	6,392 (39.5)	16,196	4.21	5,892 (43.0)	13,691	4.01	4,863 (45.4)	10,722	4.27
7 or more				773 (38.4)	2,012	3.00	4,247 (39.3)	10,816	3.81	4,962 (42.2)	11,767	3.78	5,894 (45.9)	12,841	3.58
Total offences on day of index offence															
One	34,317 (27.4)	125,215	4.56	31,184 (33.3)	93,766	4.44	27,049 (37.2)	72,801	4.24	23,151 (40.2)	57,268	4.14	19,619 (43.3)	45,334	4.08
Two or more	533 (26.2)	2,031	4.44	481 (29.9)	1,611	3.72	419 (33.2)	1,263	3.78	366 (34.6)	1,059	3.21	320 (37.0)	865	3.29
Index offence type															
Exceeding the speed limit by less than 25km/h	28,025 (29.5)	94,982	4.44	26,026 (43.3)	60,163	4.32	22,664 (47.3)	47,889	4.18	19,460 (50.9)	38,207	4.01	16,498 (54.2)	30,446	3.98
Failure to stop or give-way	473 (14.6)	3,236	5.64	330 (40.4)	816	4.92	227 (42.9)	529	4.96	169 (42.9)	394	4.57	134 (45.9)	292	4.01
Overtaking, lane use and U-turn offences	385 (18.3)	2,101	4.80	275 (40.0)	688	5.04	214 (41.7)	513	4.55	167 (49.9)	335	4.93	151 (53.5)	282	5.06
Signalling and headlight offences	235 (22.7)	1,037	4.56	103 (39.5)	261	4.32	84 (47.2)	178	4.22	76 (56.3)	135	5.23	39 (45.9)	85	3.68
Mobile phone offences	366 (26.1)	1,403	4.92	440 (35.2)	1,249	5.28	423 (38.8)	1,090	5.23	440 (42.8)	1,027	4.90	477 (52.8)	904	4.90
Seat, seatbelt and helmet offences	646 (23.3)	2,774	5.52	528 (44.8)	1,179	5.28	432 (48.9)	883	4.49	357 (50.1)	712	4.57	273 (52.5)	520	4.31
Failure to obey traffic lights	4,416 (21.6)	20,472	4.80	3,367 (34.9)	9,634	4.56	2,905 (39.9)	7,289	4.54	2,441 (44.2)	5,519	4.77	2,010 (46.0)	4,365	4.64
Tailgating	91 (23.9)	381	5.04	62 (40.3)	154	4.92	41 (38.7)	106	3.52	45 (45.5)	99	5.69	43 (51.8)	83	4.41
Unsafe/Unroadworthy vehicle	c	25	1.92	6 (54.5)	11	6.60	6 (60.0)	10	1.89	c	c	3.60	c	c	5.88
Careless driving	c	122	5.16	30 (31.9)	94	4.80	26 (37.7)	69	5.98	c	66	6.02	c	53	3.86
Licence, number plate or P-Plate display offences	189 (26.5)	713	5.64	132 (69.8)	189	3.96	76 (68.5)	111	2.94	37 (60.7)	61	3.48	22 (48.9)	45	2.79

**a** Demerit points for the day only, as there were no accumulated demerit points.

**b** Accumulated demerit points were used. The accumulated demerit points variable was generated by summing together the demerit points a driver received on the day of the current index offence, with any demerit points placed on their licence in the three years prior.

**c** Some cell counts have been supressed due to low cell counts. This has been done to maintain data confidentiality.

### Factors associated with recidivism: Driver characteristics

Table 3 shows the results of the Cox proportional hazards modelling used to investigate associations between driver characteristics and recidivism within 12 months of each index offence. If hazard ratios are below one, this means the time to reoffending was longer and therefore the risk lower. If hazard ratios are above one, this means the time to reoffending was shorter and therefore the risk higher. Statistically significant differences between males and females were only identified following the first and second offences. Time to reoffence in the following 12 months was longer for females following the first (HR = 0.81, 95% CI 0.79–0.93) and second offence (HR = 0.93, 95% CI 0.90–0.95), compared to male drivers. There was no significant difference in time to re-offend in the following 12 months between males and females following the third, fourth and fifth offences. The number of years a driver had been licensed consistently showed a statistically significant association with the time to next infringement within 12 months. Hazards ratios were greater amongst drivers who had held their licence a shorter period of time. For example, for drivers licenced less than one year, hazard ratios ranged from 1.49 (95% CI 1.43–1.55) to 3.53 (95% CI 2.85–4.37) when compared to drivers who had held a drivers licence 10+ years, depending on the number of previous offences. Age at first licence was also significantly associated with time to next infringement within 12 months. Median time to next infringement within 12 months was generally greater for drivers who obtained their licence at an older age. For example, for drivers who obtained their licence when they were aged 45+ years, hazard ratios ranged from 0.91 (95% CI 0.86–0.96) to 0.88 (0.83–0.92) when compared to drivers who obtained their drivers licence when they were aged 19.5–24 years. The association between demerit points and deterrence was also statistically significant. Following a first offence, receiving more than one demerit point had a positive deterrent effect in the following 12 months. Notably, however, receiving four or more demerit points did not necessarily have a greater deterrent effect. This can be evidenced by the greatest effect being observed for offences with three demerit points. In terms of accumulated demerit points, the results were very mixed, with no clear pattern evident of the effect on subsequent reoffending in the following 12 months (Table 3).

**Table 3:** Association between driver characteristics, demerit points and driver reoffending within 12 months.

Variable	One to two	Two to three	Three to four	Four to five	Five to six
	Event; Total (% censored)	Event; Total (% censored)	Event; Total (% censored)	Event; Total (% censored)	Event; Total (% censored)
	34,850; 127,230 (72.6)	31,665; 95,369 (66.8)	27,468; 74,056 (62.9)	23,517; 58,322 (59.7)	19,939; 46,194 (56.8)
	**HR (95% CI)**	**HR (95% CI)**	**HR (95% CI)**	**HR (95% CI)**	**HR (95% CI)**
Sex					
Male	Ref	Ref	Ref	Ref	Ref
Female	**0.81 (0.79–0.83)**	**0.93 (0.90–0.95)**	1.00 (0.98–1.03)	1.00 (0.98–1.03)	1.01 (0.98–1.04)
Age at first licence					
19.5–24	Ref	Ref	Ref	Ref	Ref
25–34	0.99 (0.96–1.03)	0.98 (0.95–1.01)	0.99 (0.95–1.02)	0.98 (0.95–1.02)	1.00 (0.96–1.04)
35–44	1.04 (1.00–1.07)	0.96 (0.93–1.00)	**0.95 (0.91–0.98)**	**0.94 (0.91–0.98)**	0.97 (0.93–1.01)
45+	**0.89 (0.85–0.92)**	**0.89 (0.86–0.93)**	**0.88 (0.84–0.92)**	**0.88 (0.83–0.92)**	**0.91 (0.86–0.96)**
Years licenced at index offence					
Less than 1	**1.49 (1.43–1.55)**	**1.96 (1.86–2.05)**	**2.37 (2.19–2.58)**	**2.82 (2.47–3.23)**	**3.53 (2.85–4.37)**
1–4	**1.20 (1.16–1.24)**	**1.46 (1.41–1.50)**	**1.53 (1.48–1.59)**	**1.57 (1.51–1.63)**	**1.54 (1.47–1.62)**
5–9	**1.14 (1.10–1.19)**	**1.23 (1.20–1.27)**	**1.24 (1.20–1.27)**	**1.26 (1.23–1.30)**	**1.24 (1.20–1.28)**
10+	Ref	Ref	Ref	Ref	Ref
Licence type					
Car only	Ref	Ref	Ref	Ref	Ref
Car and heavy vehicle	1.04 (0.99–1.09)	**1.08 (1.03–1.14)**	**1.04 (0.99–1.10)**	0.98 (0.93–1.04)	1.02 (0.96–1.09)
Car and motorbike	1.03 (0.98–1.08)	1.03 (0.98–1.08)	**1.07 (1.02–1.13)**	1.00 (0.95–1.06)	1.00 (0.94–1.06)
Car, heavy vehicle and motorbike	0.92 (0.84–1.00)	0.96 (0.88–1.04)	1.02 (0.94–1.12)	0.92 (0.83–1.01)	0.99 (0.90–1.10)
Demerit points on day of index offence^a^					
One	Ref				
Two	**0.62 (0.56–0.69)**				
Three	**0.82 (0.80–0.84)**				
Four or more	**0.62 (0.52–0.74)**				
Accumulated demerit points^b^					
1–2		Ref	Ref	Ref	Ref
3–4		**0.92 (0.90–0.94**)	0.97 (0.94–1.00)	0.96 (0.93–1.00)	1.00 (0.96–1.04)
5–6		**0.84 (0.81–0.87)**	0.99 (0.95–1.02)	1.03 (0.99–1.07)	1.08 (1.04–1.12)
7 or more		1.06 (0.98–1.14)	**0.94 (0.90–0.98)**	**0.95 (0.91–0.99)**	1.03 (0.98–1.07)
Total offences on day of index offence					
One	Ref	Ref	Ref	Ref	Ref
Two or more	**1.41 (1.21–1.64)**	**0.88 (0.81–0.97)**	**0.90 (0.81–0.99)**	**0.87 (0.78–0.96)**	**0.84 (0.75–0.94)**

*ABBREVIATIONS*: HR = hazard ratio; CI = confidence interval

^a^ Demerit points for the day only, as there were no accumulated demerit points.

^b^ Accumulated demerit points were used. The accumulated demerit points variable was generated by summing together the demerit points a driver received on the day of the current index offence, with any demerit points placed on their licence in the three years prior.

Significant hazard ratios are shown in bold.

### Factors associated with recidivism for specific offence types following each index offence

The next series of Cox proportional hazards models were stratified by offence type, this time not capturing demerit points.

#### Recidivism following the first offence

Table 4 provides the results of the Cox proportional hazards models used to examine factors associated with recidivism within 12 months following the first index offence for specific offence types. Gender was consistently found to be associated with time to re-offence within 12 months following the first offence. Females had a lower risk of reoffending in the 12 months that followed, for all offence types, when compared to males. The length of time a driver had held their drivers’ licence was also found to be associated with reoffending following the first offence. For all offence types, drivers who had been licenced less than one year had a greater risk of reoffending in the 12 months that followed when compared to drivers who had been licenced 10+ years. Indeed, for many offences, the risk of reoffending within 12 months was greater the shorter the length of time a driver had been licenced. This was particularly true following infringements for speeding below 25km/h, failure to obey a traffic signal, failure to stop or give way and mobile phone use. Age at first licence was also found to be associated with reoffending within 12 months following the first offence for some offence types. Drivers aged 45+ years when they first obtained their drivers licence and whose first infringement was for speeding below 25km/h offences, failing to stop or give way offences, seat, seat belt and helmet offences and overtaking, lane use or U-turn offences had a lower risk of reoffending in the 12 months that followed their first index offence when compared to drivers licenced prior to age 25 years. Licence type and total number of offences on the day of the index offence were not generally found to be associated with time to next infringement within 12 months across each of the offence types, with only a small number of statistically significant results observed and no clear pattern evident (Table 4).

**Table 4:** Association between driver characteristics, demerit points and driver reoffending within 12 months following the first offence, by offence type.

Variable	Speeding below 25km/h	Failure to obey traffic signal	Failure to stop or give way	Seat, seatbelt and helmet offences	Overtaking, lane use and U-turn offences	Mobile phone offences
	Event; Total (% censored)	Event; Total (% censored)	Event; Total (% censored)	Event; Total (% censored)	Event; Total (% censored)	Event; Total (% censored)
	28,025; 94,974 (70.5)	4,416; 20,470 (78.4)	473; 3,236 (85.4)	646; 2,774 (76.7)	385; 2,101 (81.7)	366; 1,403 (73.9)
	**HR (95% CI)**	**HR (95% CI)**	**HR (95% CI)**	**HR (95% CI)**	**HR (95% CI)**	**HR (95% CI)**
Sex						
Male	Ref	Ref	Ref	Ref	Ref	Ref
Female	**0.84 (0.82–0.87)**	**0.76 (0.71–0.81)**	**0.63 (0.52–0.76)**	**0.69 (0.59–0.82)**	**0.51 (0.41–0.63)**	**0.79 (0.63–0.98)**
Age at first licence (years)						
19.5–24	Ref	Ref	Ref	Ref	Ref	Ref
25–34	1.02 (0.99–1.06)	1.03 (0.93–1.14)	**0.75 (0.58–0.97)**	0.82 (0.66–1.01)	0.88 (0.67–1.16)	0.71 (0.48–1.05)
35–44	**1.09 (1.06–1.14)**	0.99 (0.89–1.10)	0.79 (0.60–1.03)	0.87 (0.69–1.09)	**0.72 (0.53–0.96)**	0.70 (0.46–1.07)
45+	**0.93 (0.89–0.97)**	0.91 (0.81–1.02)	**0.53 (0.38–0.75)**	**0.72 (0.54–0.97)**	**0.64 (0.44–0.93)**	0.74 (0.45–1.22)
Years licenced at index offence						
Less than 1	**1.43 (1.37–1.50)**	**1.72 (1.56–1.90)**	**2.09 (1.29–3.38)**	**1.47 (1.01–2.16)**	**1.70 (1.08–2.68)**	**2.82 (1.86–4.27)**
1–4	**1.15 (1.10–1.19)**	**1.35 (1.23–1.48)**	**1.81 (1.13–2.89)**	1.37 (0.95–1.99)	1.25 (0.80–1.96)	**2.10 (1.45–3.05)**
5–9	**1.08 (1.04–1.13)**	**1.29 (1.16–1.42)**	**1.89 (1.14–3.14)**	1.18 (0.79–1.77)	1.25 (0.76–2.07)	**1.90 (1.34–2.70)**
10+	Ref	Ref	Ref	Ref	Ref	Ref
Licence type						
Car only	Ref	Ref	Ref	Ref	Ref	Ref
Car and heavy vehicle	0.99 (0.93–1.05)	**1.39 (1.21–1.61)**	1.18 (0.78–1.79)	0.80 (0.60–1.06)	1.02 (0.64–1.61)	1.32 (0.84–2.08)
Car and motorbike	0.99 (0.94–1.04)	**1.26 (1.08–1.47)**	0.86 (0.51–1.44)	1.17 (0.87–1.57)	**0.51 (0.30–0.88)**	1.51 (0.89–2.55)
Car, heavy vehicle and motorbike	**0.88 (0.81–0.97)**	0.82 (0.59–1.16)	1.02 (0.45–2.29)	0.97 (0.61–1.54)	1.21 (0.66–2.23)	1.12 (0.41–3.03)
Total offences on day of index offence						
One	Ref	Ref	Ref	Ref	Ref	Ref
Two or more	0.99 (0.90–1.08)	1.05 (0.72–1.54)	0.39 (0.10–1.58)	**0.53 (0.34–0.84)**	1.02 (0.45–2.28)	1.11 (0.59–2.09)

*ABBREVIATIONS*: HR = hazard ratio; CI = confidence interval.

Significant hazard ratios are shown in bold.

#### Recidivism following the second offence

Table 5 provides the results of the Cox proportional hazards models used to examine factors associated with recidivism within 12 months following the second index offence, for specific offence types. Again, females generally had a lower risk of reoffending in the 12 months that followed the date of their second index offence, with the exception of seat, seatbelt and helmet index offences and overtaking, lane use and U-turn index offences, where, although the hazards ratios indicated a lower risk of reoffending, the results were not significant. The length of time a driver had held their licence also had an association with reoffending in the 12 months that followed the second offence, across all six offence types examined. Drivers licenced less than one year had a significantly greater risk of reoffending in the 12 months that followed the second offence, when compared to drivers licenced 10+ years. Indeed, for drivers whose second index offence was for speeding below 25km/h or failure to obey a traffic signal, all hazard ratios were statistically significant and showed a pattern that for each additional length of time licenced, the drivers in that group had a lower risk of reoffending in the following 12 months. The same pattern was also observed for drivers whose second offence was a seat, seat belt or helmet offence, an overtaking, lane use or U-turn offence or a mobile phone offence, although not all results were statistically significant. Receiving an infringement for more than one offence type on the day of the index offence was not significantly associated with recidivism in the 12 months that followed, with the exception of drivers whose most serious offence type was a speeding below 25km/h offence. For these drivers, receiving infringements for multiple offences on the same day appeared to have a deterrent effect. Age at first licence and licence type were not generally found to be associated with time to next infringement within 12 months across each of the offence types, with only a small number of statistically significant results (Table 5).

**Table 5:** Association between driver characteristics, demerit points and driver reoffending. Within 12 months following the second offence, by offence type.

Variable	Speeding below 25km/h	Failure to obey traffic signal	Failure to stop or give way	Seat, seatbelt and helmet offences	Overtaking, lane use and U-turn offences	Mobile phone offences
	Event; Total (% censored)	Event; Total (% censored)	Event; Total (% censored)	Event; Total (% censored)	Event; Total (% censored)	Event; Total (% censored)
	26,449; 75,746 (65.1)	3,501; 13,162 (73.4)	304; 1,413 (78.5)	512; 1,642 (68.8)	245; 968 (74.7)	351; 1,298 (73.0)
	**HR (95% CI)**	**HR (95% CI)**	**HR (95% CI)**	**HR (95% CI)**	**HR (95% CI)**	**HR (95% CI)**
Sex						
Male	Ref	Ref	Ref	Ref	Ref	Ref
Female	**0.94 (0.92–0.97)**	**0.88 (0.82–0.94)**	**0.78 (0.61–0.99)**	0.83 (0.68–1.01)	0.79 (0.60–1.05)	**0.76 (0.61–0.96)**
Age at first licence (years)						
19.5–24	Ref	Ref	Ref	Ref	Ref	Ref
25–34	0.99 (0.95–1.02)	0.98 (0.88–1.10)	**0.78 (0.61–0.99)**	0.89 (0.71–1.11)	1.02 (0.71–1.47)	1.15 (0.84–1.58)
35–44	0.99 (0.96–1.03)	0.93 (0.83–1.05)	0.97 (0.67–1.41)	0.82 (0.63–1.05)	1.01 (0.69–1.49)	0.71 (0.49–1.05)
45+	**0.91 (0.87–0.95)**	0.92 (0.80–1.04)	1.06 (0.70–1.62)	**0.69 (0.48–0.99)**	1.12 (0.71–1.76)	0.86 (0.52–1.44)
Years licenced at index offence						
Less than 1	**1.88 (1.78–1.99)**	**2.00 (1.73–2.32)**	**2.14 (1.37–3.36)**	**1.84 (1.26–2.70)**	**1.81 (1.03–3.19)**	**2.16 (1.38–3.39)**
1–4	**1.40 (1.36–1.45)**	**1.56 (1.44–1.70)**	1.24 (0.88–1.75)	**1.54 (1.15–2.07)**	**1.71 (1.16–2.52)**	**1.46 (1.08–1.97)**
5–9	**1.20 (1.16–1.25)**	**1.22 (1.11–1.33)**	1.34 (0.94–1.93)	1.27 (0.93–1.74)	1.49 (0.98–2.25)	1.09 (0.84–1.43)
10+	Ref	Ref	Ref	Ref	Ref	Ref
Licence type						
Car only	Ref	Ref	Ref	Ref	Ref	Ref
Car and heavy vehicle	1.05 (1.00–1.12)	**1.27 (1.08–1.49)**	1.26 (0.83–1.92)	1.16 (0.89–1.53)	1.36 (0.78–2.35)	1.08 (0.71–1.64)
Car and motorbike	1.00 (0.95–1.06)	1.10 (0.93–1.31)	1.45 (0.86–2.42)	**1.39 (1.02–1.89)**	1.00 (0.62–1.61)	1.12 (0.71–1.77)
Car, heavy vehicle and motorbike	0.91 (0.83–1.00)	1.04 (0.72–1.49)	1.43 (0.70–2.93)	1.27 (0.82–1.97)	0.66 (0.21–2.08)	0.78 (0.32–1.92)
Total offences on day of index offence						
One	Ref	Ref	Ref	Ref	Ref	Ref
Two or more	**0.90 (0.82–0.99)**	0.78 (0.54–1.13)	0.91 (0.37–2.20)	0.68 (0.41–1.14)	0.22 (0.03–1.59)	1.23 (0.61–2.51)

*ABBREVIATIONS*: HR = hazard ratio; CI = confidence interval.

Significant hazard ratios are shown in bold.

#### Recidivism following the third offence

Table 6 provides the results of the Cox proportional hazards models used to examine factors associated with recidivism within 12 months following the third index offence, for specific offence types. Unlike for the first and second index offences, there were no statistically significant associations for drivers’ sex, across any of the six offence types examined. Age at first licence also did not generally have a statistically significant effect on recidivism or deterrence within 12 months, apart from drivers who had received an infringement for speeding below 25km/h offence. Amongst these drivers, those aged 45+ when they first obtained their drivers licence had a lower risk of reoffending within 12 months than those who obtained their drivers licence before age 25 years. This trend was also observed in some other offence types, but statistical significance was not reached. Again, years licenced was also found to generally have a significant effect on recidivism and deterrence within 12 months. Across all offence types, with the exception of drivers whose third index offence was for an overtaking, lane use and U-turn offence, drivers who had held their licence less than 1 year were at greater risk of re-offending in the 12 months that followed, compared to drivers who had held their licence for 10+ years. Once again, a pattern was also observed where risk decreased gradually, the longer a driver had held a licence. This pattern was observed for all offence types, however not all results were statistically significant (Table 6).

**Table 6:** Association between driver characteristics, demerit points and driver reoffending within 12 months following the third offence, by offence type.

Variable	Speeding below 25km/h	Failure to obey traffic signal	Failure to stop or give way	Seat, seatbelt and helmet offences	Overtaking, lane use and U-turn offences	Mobile phone offences
	Event; Total (% censored)	Event; Total (% censored)	Event; Total (% censored)	Event; Total (% censored)	Event; Total (% censored)	Event; Total (% censored)
	23,286; 59,875 (61.1)	2,828; 9,571 (70.5)	170; 813 (79.1)	400; 1,170 (65.8)	205; 682 (69.9)	382; 1,260 (69.7)
	**HR (95% CI)**	**HR (95% CI)**	**HR (95% CI)**	**HR (95% CI)**	**HR (95% CI)**	**HR (95% CI)**
Sex						
Male	Ref	Ref	Ref	Ref	Ref	Ref
Female	0.99 (0.97–1.02)	1.04 (0.96–1.12)	1.13 (0.81–1.58)	0.89 (0.71–1.11)	0.94 (0.70–1.28)	0.94 (0.76–1.17)
Age at first licence (years)						
19.5–24	Ref	Ref	Ref	Ref	Ref	Ref
25–34	0.99 (0.96–1.03)	1.02 (0.91–1.15)	0.96 (0.64–1.52)	0.91 (0.71–1.16)	1.09 (0.74–1.62)	1.28 (0.95–1.71)
35–44	0.97 (0.94–1.01)	0.91 (0.81–1.04)	0.88 (0.55–1.39)	0.78 (0.58–1.04)	0.96 (0.63–1.47)	1.05 (0.75–1.48)
45+	**0.91 (0.87–0.96)**	0.89 (0.77–1.03)	0.80 (0.46–1.42)	0.46 (0.29–0.75)	1.01 (0.59–1.72)	0.82 (0.47–1.42)
Years licenced at index offence						
Less than 1	**2.24 (2.05–2.44)**	**2.63 (2.04–3.39)**	**3.29 (1.37–7.88)**	**2.80 (1.56–5.00)**	2.00 (0.93–4.28)	**2.43 (1.22–4.82)**
1–4	**1.45 (1.41–1.50)**	**1.78 (1.62–1.96)**	**1.87 (1.24–2.80)**	**1.75 (1.31–2.33)**	**1.75 (1.21–2.53)**	1.23 (0.92–1.64)
5–9	**1.20 (1.16–1.23)**	**1.25 (1.14–1.37)**	1.37 (0.90–2.06)	**1.42 (1.06–1.90)**	1.33 (0.91–1.92)	1.07 (0.85–1.36)
10+	Ref	Ref	Ref	Ref	Ref	Ref
Licence type						
Car only	Ref	Ref	Ref	Ref	Ref	Ref
Car and heavy vehicle	1.02 (0.96–1.02)	**1.23 (1.02–1.48)**	1.70 (0.94–3.08)	0.84 (0.61–1.17)	**1.72 (1.06–2.82)**	0.89 (0.58–1.34)
Car and motorbike	1.04 (0.98–1.09)	**1.26 (1.05–1.50)**	1.34 (0.69–2.61)	0.98 (0.66–1.44)	1.54 (0.90–2.65)	1.10 (0.69–1.75)
Car, heavy vehicle and motorbike	0.97 (0.88–1.07)	**1.48 (1.07–2.04)**	1.91 (0.88–4.15)	1.09 (0.66–1.79)	1.36 (0.55–3.34)	0.50 (0.18–1.35)
Total offences on day of index offence						
One	Ref	Ref	Ref	Ref	Ref	Ref
Two or more	0.90 (0.81–1.00)	0.80 (0.54–1.19)	1.23 (0.32–5.23)	0.80 (0.50–1.26)	1.14 (0.42–3.07)	0.70 (0.26–1.88)

*ABBREVIATION*: HR = hazard ratio; CI = confidence interval.

Significant hazard ratios are shown in bold.

#### Recidivism following the fourth offence

Table 7 provides the results of the Cox proportional hazards models used to examine factors associated with recidivism within 12 months following the fourth index offence for specific offence types. Consistent with recidivism following the third index offence, gender was not found to have a statistically significant effect on recidivism within 12 months for any offence type. Similarly, age at first licence was also not generally found to be associated with recidivism within 12 months, following the fourth offence, with only two statistically significant results identified. Amongst drivers who received an infringement for speeding below 25km/h or failing to obey a traffic signal, those who received their licence at age 45+ years had a lower risk of reoffending in the 12 months that followed compared to drivers who were licenced below the age of 25 years. As was observed with reoffending following the first, second and third offences, the length of time a driver had been licenced was found to be associated with recidivism within 12 months across all offence types examined. Indeed, the lesser the period of time that a driver had been licenced, the greater their risk of reoffending in the 12 months that followed. In terms of licence type, there were only two significant results found, indicating this factor was generally not associated with reoffending within 12 months following the fourth index offence. The number of offences on the day of the index offence was also generally not associated with recidivism within 12 months, with the exception of drivers whose fourth index infringement was for speeding below 25km/h. Amongst these drivers, those who received infringements for other offence types on the same day had a lower risk of recidivism in the 12 months that followed when compared to drivers who only received an infringement for the speeding offence (Table 7).

**Table 7:** Association between driver characteristics, demerit points and driver reoffending within 12 months following the fourth offence, by offence type.

Variable	Speeding below 25km/h	Failure to obey traffic signal	Seat, seatbelt and helmet offences	Mobile phone offences
	Event; Total (% censored)	Event; Total (% censored)	Event; Total (% censored)	Event; Total (% censored)
	20,079; 47,660 (57.9)	2,346; 7,227 (67.5)	306; 890 (65.6)	343; 1,070 (67.9)
	**HR (95% CI)**	**HR (95% CI)**	**HR (95% CI)**	**HR (95% CI)**
Sex				
Male	Ref	Ref	Ref	Ref
Female	1.01 (0.98–1.04)	0.98 (0.90–1.07)	0.90 (0.70–1.15)	0.82 (0.66–1.03)
Age at first licence (years)				
19.5–24	Ref	Ref	Ref	Ref
25–34	1.01 (0.97–1.04)	0.93 (0.82–1.05)	0.87 (0.65–1.15)	1.07 (0.81–1.41)
35–44	0.97 (0.93–1.01)	0.95 (0.83–1.08)	0.76 (0.54–1.07)	0.84 (0.60–1.18)
45+	**0.92 (0.87–0.97)**	**0.82 (0.70–0.97)**	0.78 (0.49–1.23)	0.23 (0.09–0.71)
Years licenced at index offence				
Less than 1	**2.75 (2.38–3.19)**	**2.21 (1.43–3.41)**	**2.98 (1.08–8.23)**	**14.57 (4.56–46.57)**
1–4	**1.54 (1.48–1.60)**	**1.62 (1.44–1.81)**	**1.87 (1.37–2.56)**	1.37 (0.96–1.94)
5–9	**1.24 (1.20–1.28)**	**1.23 (1.12–1.35)**	**1.51 (1.13–2.03)**	1.16 (0.92–1.47)
10+	Ref	Ref	Ref	Ref
Licence type				
Car only	Ref	Ref	Ref	Ref
Car and heavy vehicle	0.97 (0.91–1.03)	1.15 (0.95–1.39)	1.15 (0.81–1.63)	**0.59 (0.36–0.98)**
Car and motorbike	1.01 (0.95–1.07)	0.97 (0.70–1.21)	0.65 (0.37–1.15)	0.85 (0.51–1.42)
Car, heavy vehicle and motorbike	**0.87 (0.78–0.97)**	**1.42 (1.03–1.95)**	1.07 (0.61–1.87)	0.93 (0.44–1.99)
Total offences on day of index offence				
One	Ref	Ref	Ref	Ref
Two or more	**0.88 (0.78–0.98)**	0.70 (0.45–1.09)	0.96 (0.60–1.55)	0.48 (0.12–1.95)

*ABBREVIATION*: HR = hazard ratio; CI = confidence interval.

Significant hazard ratios are shown in bold.

#### Recidivism following the fifth offence

Table 8 provides the results of the final series of Cox proportional hazards models, used to examine factors associated with recidivism within 12 months following the fifth index offence. Gender was not found to have any statistically significant effect on reoffending within 12 months. This was consistent with the pattern that emerged following the third and fourth index offences. Age at first licence was not generally found to be associated with recidivism within 12 months, with only one significant result emerging. Drivers who were aged 45+ years when they received their licence had a lower risk of reoffending in the 12 months that followed when compared to drivers licenced prior to the age of 25 years, where the fifth index offence was for speeding below 25km/h. Consistent with the patterns that emerged in the Cox proportional hazards models for the earlier index offences, length of time licenced was also found to be associated with recidivism within 12 months following the fifth index offence. The shorter the period of time a driver had been licenced, the greater was the risk of reoffending within 12 months, irrespective of offence type. No statistically significant associations were identified for licence type, irrespective of offence type. Finally, for drivers whose most serious offence was speeding below 25km/h on their fifth index offence, receiving an infringement for an additional offence had a deterrent effect when compared to drivers who only received a single infringement for the speeding offence (Table 8).

**Table 8:** Association between driver characteristics, demerit points and driver reoffending within 12 months following the fifth offence, by offence type.

Variable	Speeding below 25km/h	Failure to obey traffic signal
	Event; Total (% censored)	Event; Total (% censored)
	17,015; 37,973 (55.2)	1,899; 5,451 (65.2)
	**HR (95% CI)**	**HR (95% CI)**
Sex		
Male	Ref	Ref
Female	1.01 (0.98–1.04)	1.00 (0.90–1.10)
Age at first licence (years)		
19.5–24	Ref	Ref
25–34	1.01 (0.97–1.05)	0.95 (0.83–1.09)
35–44	0.97 (0.93–1.02)	0.92 (0.80–1.06)
45+	**0.93 (0.87–0.99)**	0.88 (0.74–1.06)
Years licenced at index offence		
Less than 1	**3.49 (2.79–4.36)**	**4.51 (2.13–9.56)**
1–4	**1.52 (1.45–1.59)**	**1.58 (1.36–1.83)**
5–9	**1.21 (1.17–1.25)**	**1.34 (1.21–1.49)**
10+	Ref	Ref
Licence type		
Car only	Ref	Ref
Car and heavy vehicle	1.03 (0.96–1.10)	0.96 (0.77–1.20)
Car and motorbike	0.98 (0.92–1.05)	1.03 (0.83–1.27)
Car, heavy vehicle and motorbike	0.98 (0.88–1.09)	0.99 (0.66–1.48)
Total offences on day of index offence		
One	Ref	Ref
Two or more	**0.88 (0.78–0.99)**	0.62 (0.38–1.00)

*ABBREVIATION*: HR = hazard ratio; CI = confidence interval

Significant results are shown in bold

## Discussion

### Summary and key findings

A number of driver characteristics were associated with recidivism and time to reoffending within 12 months following a traffic offence. Irrespective of offence type, male drivers were quicker to reoffend within 12 months than female drivers, amongst those with only one or two previous offences. These differences, however, were not observed in drivers with more than two previous infringements. These findings are in some respects consistent with existing international literature. Female drivers perceive levels of risk to be higher than male drivers, with male drivers subsequently showing higher rates of risky driving behaviour [51–53]. If drivers perceive levels of risk to be higher, we may reasonably expect they would be less likely to reoffend following a traffic infringement. The disappearance of the gender differences in latter offences may be explained by the idea of some offenders being incorrigible [54]. For drivers with extensive offending histories, it is likely that, regardless of gender, we are seeing a group of offenders for whom their behaviour is not amenable through the use of traffic infringements.

Irrespective of offence type, newly licenced drivers consistently had a higher risk of reoffending within 12 months, when compared with drivers who had held their licence for an extended period of time. Previous studies have found that young, new drivers are one of the riskiest groups of drivers on the roads, and can be resistant to deterrence by legal sanctions [55–58]. However, the model used in this study adjusted for age, indicating that for any age group, those drivers who have had their licence for the least amount of time have a shorter time to next infringement. This may be the result of a combination of two factors. Firstly, inexperienced drivers may not recognise the risks associated with particular behaviours, such as mobile phone use whilst driving, and thus may be more inclined to perform such behaviours. Secondly, inexperienced drivers may be less skilled than more experienced drivers, meaning they may be more likely to make errors while driving, such as failing to stop or give way, resulting in them receiving traffic infringements.

Driver sex and years licenced are not factors that can be modified to decrease risk of further offending. They do, however, indicate groups of drivers for whom it may be beneficial to develop strategies that aim to prevent repeat offending.

Existing research has suggested that some groups of drivers, such as newly licenced drivers displaying P-plates, feel that police specifically target them for enforcement [59]. Whilst targeting of enforcement may potentially be a factor in the high levels of [captured] recidivism seen in some groups in the study, such as amongst drivers licenced less than one year, the results do not support this to be the primary factor. The two most prevalent offence types (speeding at less than 25km/h over the speed limit and failure to obey a traffic signal) are most widely enforced in Victoria via the use of an automated camera system. Police discretion is therefore unlikely to be a factor that explains the greater levels of recidivism within 12 months observed amongst drivers with less experience.

Licence type was not generally found to have a significant influence on recidivism and time to reoffending. Existing research has shown mixed results on whether drivers’ behaviour differs depending on whether they are operating a car or a motorcycle. A study by Rowden et al found evidence to suggest that individuals ride motorcycles in a less aggressive and less risky manner compared to when they are driving a car, due to the increased vulnerability associated with motorcycle riding [60]. In contrast, a study by Horswill and Helman found that motorcycle riders generally travelled at higher speeds and took more risks when overtaking and changing lanes, when compared to car drivers [61]. The current study was not able to confirm either perspective. This was for two primary reasons. Firstly, the data used did not contain information on the type of vehicle a person was operating at the time of an offence. Thus, comparisons could not be drawn between differences associated with the operation of specific vehicle types. Secondly, given drivers in this study all held a car licence at a minimum, with potential other licence types held in addition to a car licence, they may be in some respects different to individuals who only have a motorcycle licence. Further research may seek to explore these differences.

The direction of associations between demerit points and deterrence were perhaps the most surprising and interesting results that emerged from this study. Following the first offence, drivers who received multiple demerit points were less likely to reoffend in the year that followed, when compared to drivers who received only a single demerit point. This suggests that demerit points issued to drivers following a first offence may achieve deterrence. Unfortunately, however, the subsequent influence of accumulated demerit points was not quite as promising. For drivers with multiple prior offences and a high number of accumulated demerit points, demerit points did not have a statistically significant deterrent effect. It may be possible we are seeing an emboldening effect here, where, as a result of receiving an infringement for a traffic offence, drivers were encouraged, as opposed to discouraged to perform further offences [62]. Similarly, Pogarsky & Piquero suggested that when drivers receive a punishment, they may have a higher risk of reoffending soon after, as a result of “gamblers fallacy”, where they have a belief the risk of being apprehended again within quick succession is small [63]. The findings relating to demerit points are not consistent with the results from Haque, who found that a higher number of accumulated demerit points had a positive influence on deterrence [40]. It is notable that there were some differences in the methodological approach taken by Haque [40]. Firstly, the previous study included a longer follow up time period of three years, compared to the one year undertaken in the current study [40]. Secondly, the time to event approach taken here, that required drivers to only have ever received one infringement, censoring them if they did not receive a second infringement within 12 months, was different to that taken in the Haque study [40]. Haque’s study required that all drivers had to have received at least two infringements, with the second coming within three years of the first. Indeed, for this reason, Haque only included drivers who had held their licence a minimum of three years [40]. No minimum period for holding a licence was implemented in this study. This is of particular note, given the current study showed higher levels of deterrence in more experienced drivers. Thus, the differences in study findings observed may have been a result of methodological differences, in addition to or rather than changes that have occurred in use of the road network and sanctioning over the last 30+ years.

### Study implications

Overall, despite the current traffic infringements system in Victoria appearing to have a positive influence on the behaviour of some drivers, there remain groups of drivers for whom the current infringements system may not be achieving deterrence, with the most notable being newly licenced drivers. Furthermore, the current demerit point system appeared effective for first-time offenders but not for repeat offenders. To achieve a reduction in the number of serious road crashes that result from drivers performing illegal driving behaviours, it is essential that the sanctioning system is flexible in responding to different groups of offenders. This could involve the use of mandatory driver education, or greater use of technology solutions such as speed limiting systems, in addition to traffic infringements, targeted at high-risk groups such as repeat offenders and novice drivers, to reduce reoffending in these groups.

### Study strengths

The current study has many strengths that make it valuable in enhancing understandings of the Victorian infringements system. First, the study used a very large population of drivers. Second, with approximately 13 years of data available, the study was a comprehensive longitudinal analysis. Third, the range of variables and the depth of information available meant the study was able to take into account a range of potential risk factors for recidivism. Fourth, given some of the drivers whose data is included in the extract for this study were of quite advanced age by the end of the study period, the inclusion of older drivers in research is highly valuable. Existing research has tended to focus on younger drivers, with less focus on the mature driver population. This is despite the number of older drivers increasing [64, 65], a pattern that is expected to continue into the future [66].

### Study limitations

There are some limitations that must be considered when interpreting the results. The most notable limitation is that the results are not necessarily generalizable beyond the jurisdiction of Victoria, Australia. Enforcement of traffic rules and regulations is the responsibility of individual jurisdictions. In Australia, there are eight states and territories. Each runs independently of one another on issues of licensing and infringements. Whilst there are many similarities between jurisdictions that may mean the results here are relevant, other jurisdictions should examine the degree to which these results may be applicable to their local situation.

A second limitation that should be acknowledged relates to the group of drivers for whom data was drawn upon for use in this study. Data were only available for drivers born prior to 31 December 1974 and licenced between 8 July 1994 and 21 May 2016. Many drivers who were born on or prior to 31 December 1974 would have already held their licence prior to July 1994, and were therefore not eligible for inclusion in the study, as their full infringement history was not available. As a result, the study sample is relatively aged and has a larger number of individuals who received their licence at an older age (resulting in an overrepresentation of females) than would generally be expected in the Victorian driving population. However, as noted above, an examination of older drivers may be considered advantageous.

### Areas for future research

The current study has provided valuable information in relation to traffic infringements and reoffending. There are however a number of other areas where further research may be valuable in further developing knowledge in this area. First, research could focus on drivers with a high number of demerit points, such that they are close to losing their licence, or alternatively drivers who have reached 12-point demerit points (which is the demerit point limit in a three-year period for drivers on a full licence) and have elected to take an extended demerit point period. This extended demerit point period enables them to keep driving, but sees them have their licence suspended for double the suspension length they would have ordinarily received, if they reoffend within the following year. Such research may help us understand the factors that may be acting as barriers to deterrence amongst some of the most serious traffic offenders.

Second, the current study focussed on one type of sanction administered to drivers for traffic offences in Victoria, this being infringements. Drivers can also receive other sanctions for more serious offending, including licence suspension, and even a period of imprisonment. The results presented in this study are therefore not indicative of time to reoffending following these other forms of sanctioning. Future research could consider time to traffic reoffending in Victoria following licence loss or a period of imprisonment for a traffic offence. Furthermore, future research could draw comparisons between different types of sanctions, to gain an understanding of what might be the most effective strategy or strategies for responding to illegal driving behaviours. Such a study may be beneficial not only in Victoria, where the current study was undertaken, but also in other jurisdictions, considering local circumstances. Further research can also include comparisons between jurisdictions where approaches in responding to traffic offences vary. Such an approach may provide an opportunity to identify best practices and learn from the experiences of other jurisdictions.

Third, between 1994 and 2016, which, was the time period considered in the current study, vehicle technology underwent substantial change. Features such as Autonomous Emergency Braking (AEB) [67], Blind Spot Monitoring (BSM) [68], Lane Departure Warning (LDW) [69] and Traffic Sign Recognition (TSR) [70] became available in vehicles. Widespread use of smartphones also emerged across the study period [71]. In addition, technology that specifically seeks to respond to drivers who are repeat traffic offenders also came to be more widely used. This includes, for example, the use of alcohol interlock devices for drink drivers [72]. Future research that tracks the rollout of new and emerging technologies may prove valuable in examining the effects these technologies have on the performance of illegal driving behaviours and reoffending.

Finally, the analytical approach taken in the current research approach proved to be effective in addressing the aims of the study. However, to further enhance the longitudinal approach taken, future research could also multilevel modelling, to further explore traffic offending and recidivism. Multilevel models are useful for examining events that can happen repeatedly. Data are arranged in a hierarchy, with level one, which is the occurrence of the event occurring, being nested in level two, which is the individual the event relates to [73]. Such a study may enable a detailed examination of the factors that underlie an individual moving in and out of traffic offending behaviour, through for example, experience of employment and unemployment or good health and poor health. The data set used in the current study did not provide this type of information, thus new data sources would need to be explored.

## Conclusions

In conclusion, the current study shows that whilst infringements for some groups of drivers are associated with a lower risk of subsequent traffic offending within 12 months, infringements are not equally effective in deterring all groups of drivers. Rethinking how to respond to repeat offenders is a crucial step to achieving greater safety on the roads. Introducing new ways of responding to some groups of drivers will no doubt receive resistance from some members of the community. Despite this, it is important to recognise the most important goal is achieving safety for all road users. Finding the best approaches to responding to road user behaviour provides the greatest opportunities to make progress towards decreasing the number of road crashes, and ultimately deaths, that result from risky and illegal driving behaviours.
